# Geopolitical Issues and Responsibilities of Medical and Scientific Journals

**DOI:** 10.5041/RMMJ.10177

**Published:** 2015-01-29

**Authors:** Richard Horton

**Affiliations:** Editor-in-Chief, The Lancet, London, UK

**Keywords:** Medical journals, scientific journals, geopolitics

**Figure:** The video of this lecture is available online on the Rambam Maimonides Med J website.

## Video Transcript

### OPENING WORDS

I am extraordinarily humbled and proud to be here. I want to thank you, Professor Skorecki, and you, Professor Beyar, for your courage, your openness, and your generosity of spirit in inviting me here. I want to thank all those whom I have met this week for their kindness, their encouragement, and their insights.

I have learned a great deal during the past three days: Rambam, as a model for partnership between Jews and Arabs; Rambam as a center offering an open hand to the people of Palestine; and Rambam as a place with a unique vision for a peaceful, productive, and diverse future among peoples. “Rambamism,” as somebody put it to me this week.

Today is an opportunity for me, and I believe for us together. But before I talk about that opportunity, I need to set the record straight. First, I deeply regret the completely unnecessary polarization caused by the recent publication of a letter by Paola Manduca and colleagues. Irrespective of our intentions, this outcome was not my goal. Second, and contrary to some incomplete accounts of an interview I gave to one journalist, I was horrified by the offensive video forwarded by two of the authors of that letter. The world view expressed in that video is abhorrent and must be condemned, and I condemn it unreservedly. I have made my view very clear to those two individuals. Third, I will be publishing these words in *The Lancet* next week.

Let me also add: *The Lancet* is, always was, and, under my leadership, always will be one hundred percent open, indeed more than that, welcoming of research and work submitted to us from colleagues and friends in Israel. I have received, these past two months, some vile accusations about me and about my views concerning this country and its people. Let me make it absolutely clear to you: anyone who makes those claims simply does not know me, does not know anything about my life, my family, or my values.

For reasons that I find quite hard to explain and to understand myself, I have a special feeling for this land on which Israel and Palestine and the wider Arab world and their peoples live. This feeling is why over the past 7 years I have come back again and again, often several times each year, to this region. Being here this week provides me with an opportunity to open a new chapter in that relationship.

### INTRODUCTION

I am not going to make an argument this morning. I simply want to tell a story about how and why we have arrived at where we are today.

**Figure 1.**
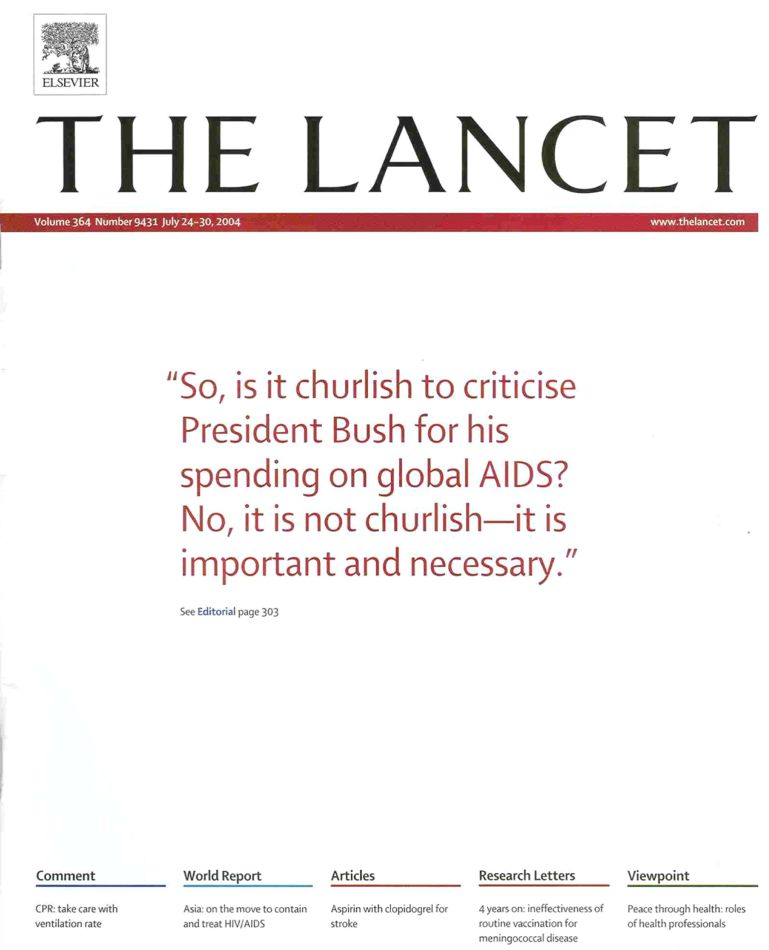
**Cover of a 2004 Issue of *The Lancet*.**

Editors can make their readers very angry. A few years ago we published these words on the front cover of *The Lancet* (Figure 1). It was about President Bush’s commitment to defeat the global AIDS epidemic. His investment in AIDS was a much admired commitment, but it took a particularly negative and critical perspective on sexual and reproductive health. When we wrote an editorial about President Bush’s polices, we said this: “So, is it churlish to criticize President Bush for his spending on global AIDS? No, it is not churlish—it is important and necessary.”^1^

Here was one response: “Is it churlish to tell the Editor that his smug, arrogant, sanctimonious new Lancet cover sucks?” Well, Dr. Warren in North Vancouver, I’m very sorry. We upset you. It’s an illustration of how editors can fail to make the best of their statements and intentions.

The subject of this talk concerns Geopolitical Responsibilities. Right now, the world is passing through an extraordinary crisis. It is not only a health crisis. The CDC (Centers for Disease Control and Prevention) in Atlanta has estimated that if we do nothing, by the end of January (2015), there will be up to 1.4 million cases of Ebola in and around West Africa. We have called that situation “a failure of international collective action.”^2^ But it is far more than that.

Just a week ago I received an email from a colleague at USAID who wrote: “We very much agree on the need to improve the resources and capabilities of the multilateral agencies to deadly outbreaks like Ebola, and this will be covered…as part of a global security agenda.” Right now, in West Africa and elsewhere (since the first case of Ebola was diagnosed in the United States just this week), health has become a hugely important geopolitical issue, as well as a responsibility, that we share together.

### THE GEOPOLITICAL IMPORTANCE OF HEALTH

We know this. It is not new. And in the fields of epidemiology and public health, we have understood over many years the centrality of politics to health. Johan Mackenback wrote just a few years ago:

“…human health and disease are the embodiment of the successes and failures of society as a whole, and the only way to improve health and reduce disease is by changing society by, therefore, political action.”^3^

He went so far as to say that politics “is public health’s biggest idea.”^3^

Coming closer to home in the United Kingdom, let me turn to a gastroenterologist who is long dead now, but who, in the middle of his academic life, changed trajectories. John Ryle founded the United Kingdom’s first Department of Social Medicine at the University of Oxford. He wrote these words in his reflections on his change of trajectory—his mid-life crisis in thinking about the direction of his work:

Ethical values cannot be measured as the death rate in infancy…but that there is a relationship between the revelations of vital statistics and human responsibility in a modern society we can hardly dispute.”^4^

**Figure 2.**
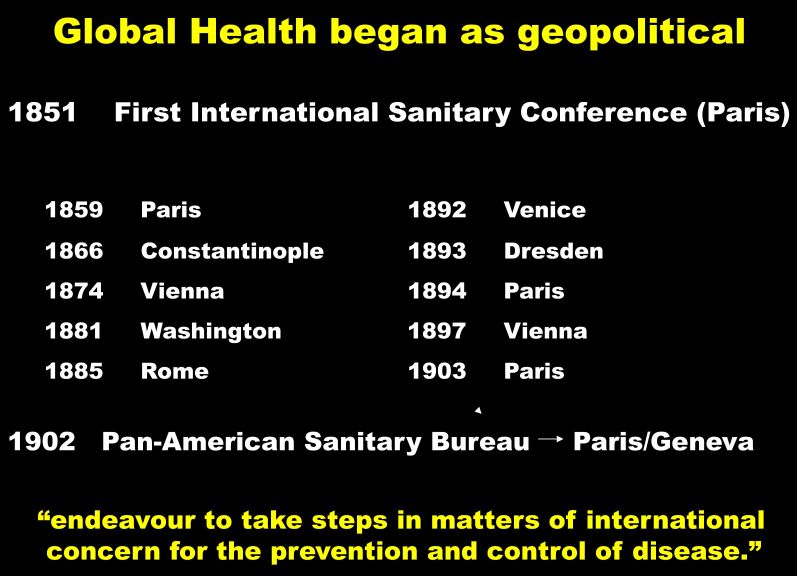
**Global Health Began as Geopolitical.**

Here was his definition of social medicine: our “human responsibility.” Globalize that idea, and in the very founding of the World Health Organization, from the first Sanitary Conference held in Paris in 1851, through a series of meetings that took place over the next century (Figure 2), nations understood the geopolitical importance of health and the vital need to come together, to work together, to improve health. Countries endeavored to take international action for the prevention and control of disease.

That, very briefly, is a global story about health. What about the same story through the lens of one medical journal?

### GEOPOLITICS, HEALTH, *THE LANCET*, AND ISRAEL

*The Lancet* seeks to be a global medical and scientific journal. But we are not fully proud of its beginnings. *The Lancet*, in its opening editorial by Thomas Wakley on October 5, 1823, described its purpose, part of which was to speak “to colonial practitioners”^5^: Great Britain’s (some would say) not so great past as an empire. And we distinguished in that opening editorial between two parts of the world: England and the rest of the civilized continent. I hope we do a little better today. Let me give you one example of *The Lancet*’s commitment to some of these international issues, which have inescapably critical political dimensions.

**Figure 3.**
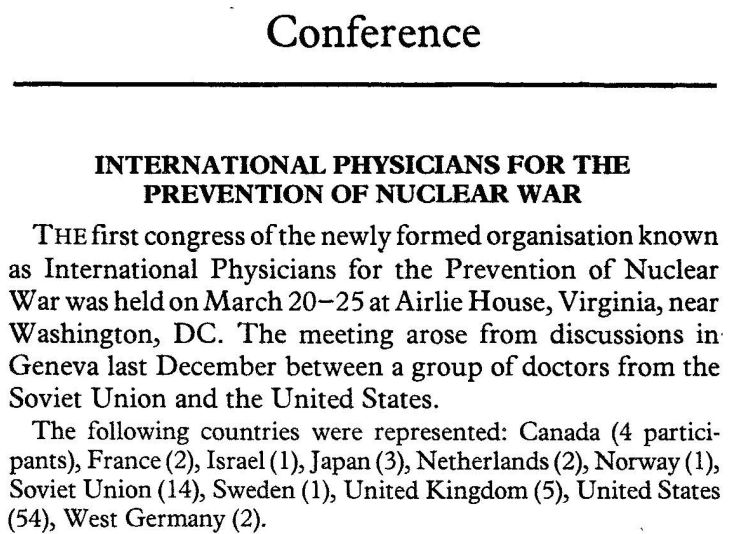
**Report on the First Congress of the International Physicians for the Prevention of Nuclear War.**

The International Physicians for the Prevention of Nuclear War (IPPNW) held its very first congress in 1981. Please note: there is Israel, your political commitment was represented at that very first meeting (Figure 3). This movement against nuclear war, created by two physicians, one in the United States and one in what was then the Soviet Union, received incredible criticism for being an alleged vehicle of Soviet propaganda. But, especially thanks to the efforts of one individual, Bernard Lown, IPPNW won the Nobel Peace Prize in 1985. And since you were part of this initiative, I hope you claim the Nobel Peace Prize, at least partly, as a victory for Israel’s commitment to one aspect of the politics of health. Here is what *The Lancet* said at the time of the award of the Nobel Peace Prize to IPPNW:

“The political implications of the campaigns voiced by IPPNW and other professional bodies…are inescapable. If the medical profession flinches from all conceivably political tone…then its influence will be weakened.”^6^

That message was taken to heart by one nation in particular, and I think you may guess which nation that is. Because Israel, a year later, created its own chapter of IPPNW, and here was the oath that was given at a meeting of the Israeli Association of Physicians for the Prevention of Nuclear War, on January 29, 1986:

“I pledge, in order to protect the welfare of the human being, never to use nuclear energy for destructive purposes and I shall devote my life as a physician to fight against disease, poverty, ignorance, and nuclear war.”

Dr Kahan, who was Chairman of the Israel Association for the Prevention of Nuclear War, wrote this in *The Lancet* in 1986:

“Israeli journalists, authors, social workers, parents, kibbutz members, and many others, directly influenced by IPPNW’s medical prescription campaign and IPPNW’s message to patients, understood the need to join physicians in this crusade against nuclear destruction. Their answer was the foundation of the Israel Committee for the Prevention of Nuclear War in 1986.”^7^

As a nation, a people, and as health professionals, Israel and Israelis have been at the forefront of leadership in putting health and medicine—our profession—on the frontlines of these geopolitical concerns.

### THE IMPACT OF POLITICS ON MEDICINE AND SCIENCE

I joined *The Lancet* in the early 1990s and my first exposure to the relationship between health and war came with an invitation from two people in Croatia who edited the *Croatian Medical Journal*, Ana and Matko Marušić. They invited me (after the war in Croatia) to visit their country, to meet in Zagreb, to travel to the city of Vukovar in the northeast, which was utterly destroyed during the war, and then to travel south to Split and Dubrovnik. In traveling around that part of my own continent, understanding the importance of health and medicine to a society in conflict, I saw how important it could be for a country to investigate where it is today and what its future might be by analyzing its attitudes to health. I did not know what to do with that feeling of a connection between health and one’s identity until I met a scientist named Jennifer Bryce, who was at that time working at UNICEF. Jennifer asked a decisive and troubling question. “Richard, you claim to be the editor of an international medical journal. What do you know about children’s health globally?”

I had to admit (I was previously an adult physician) that I knew very little about children’s health. And Jennifer replied, “You should be ashamed of yourself!” It was Jennifer who then took my hand, quite literally, through a process lasting over two years, with the eventual publication of our first series in global health (on child survival). The work we did together was a revelation to me. At the end of that series—four papers describing the human toll of child mortality in the world—we asked what could be achieved if the world took a different course. Instead of being a series of papers about *just* epidemiology and science, the final paper was a call for action.

“We, a group of concerned scientists and public-health managers, call on: WHO, UNICEF, the World Bank, the UNDP, and their other UN partners to act on behalf of children by putting child survival at the top of their list of priorities.”^8^

These public health scientists had a political agenda. They wanted child health at the top of the world’s political priorities. And they turned their message into a campaign. It is over 10 years on from that moment now. We have had many, many similar projects: around 50 series that have tried to make the claim for the importance of the issues they have covered—not only the epidemiological or public health importance, but also the social and political importance. The conclusions that we have drawn from the decade of work in this area are that:

- Science can be a powerful catalyst for shaping and changing policy;
- partnerships between people, in whatever domain of study they might inhabit, can deliver impact;
- doctors, together with other health professionals, can trigger social action; and
- health professionals can be leaders of political, as well as clinical and public health, change.

Let me give one example. We have published four series during the past decade on noncommunicable diseases. As you know, there is a massive epidemiological transition taking place across the world. In 2011, a “Political Declaration” about the importance of non-communicable diseases was signed at the United Nations under the leadership of UN Secretary-General, Ban Ki-moon. When he stood to sign this document, it was indeed a political, not merely a scientific, health, or medical commitment.^9^

To make change, to motivate change, we sometimes have to embrace these political dimensions. Occasionally, that stance will bring us into face-to-face conflicts, in opposition to the often huge forces shaping our society.

**Figure 4.**
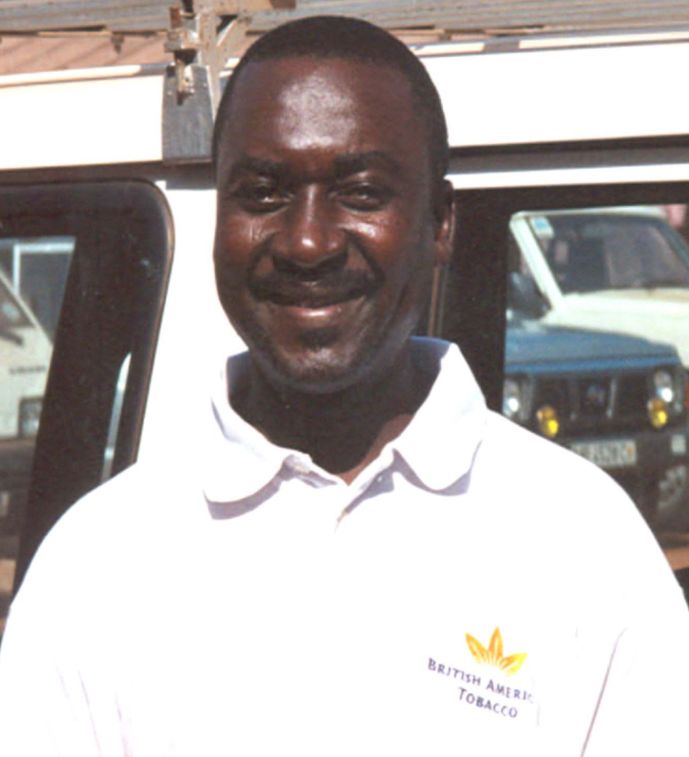
**Government Worker with British American Tobacco Shirt.**

When I first visited Ethiopia, this man very kindly took us to the south-west regions of the country. Now, what is the clinical sign? It’s hard to see—but here it is (Figure 4). He works for the Ministry of Health, all of whose staff were at that time all provided with clothes from British American Tobacco.

The forces that we face in many parts of the world today, shaping health, colluding frequently in the worst interests of health, can be very powerful and require us to go beyond simply research and the delivery of health care. Those forces require us to stand on the political frontlines of health.

When we think about the context of noncommunicable diseases, about where we go after the Millennium Development Goals, whose time-bound window closes at the end of 2015, and when we contemplate a new era of sustainable development after 2015 (2016–2030), we are going to have to rethink our definitions of human development and put the idea of sustainability at its core.

**Figure 5.**
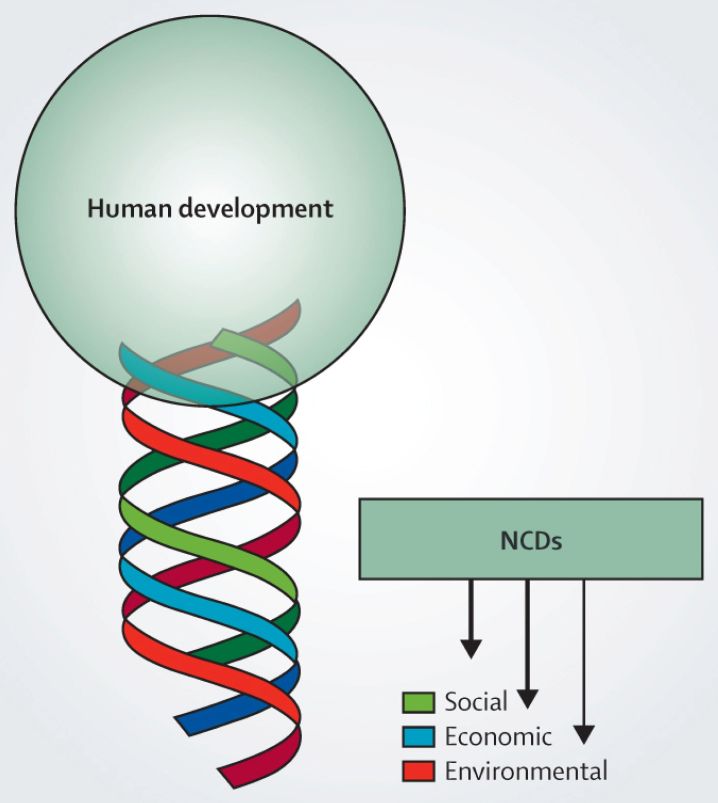
**The Effects of NCDs on the Sustainability of Human Development.** The representation of three strands of the helix that make development sustainable and the effects of noncommunicable disease (NCDs) on those strands. Courtesy of Knowledge Management and Communication, PAHO/WHO.

As for non-communicable diseases, we must begin to consider the social determinants of disease, the economic dimensions of illness, and the environmental context of health (Figure 5). And, I would insist, the political determinants of disease, illness, and health too.

In the opening slide of this lecture, I included a picture from last week’s United Nations General Assembly Climate Summit. Ten years ago I would have never considered attending the United Nations General Assembly. I am a doctor. I have no expertise or even interest in UN summitry. The General Assembly is a meeting for heads of state, not health professionals. But now, doctors and health professionals attend every year. Why? Because, for one week, in New York at the General Assembly, global politics is all about health. An enormous health program runs from the early hours of every morning through to the late evening of every day.

Why has global health become so popular as well as political? Some years ago now, we wanted to use *The Lancet* as a vehicle to draw attention to broader issues and themes in health. The first subject we chose was climate change. It’s hard to think of climate change as a health issue. Surely it is about fossil fuels, melting glaciers, and extreme weather events. But climate change is most definitely and deeply a health issue. What we sought to do was show how a multi-faculty university can help us understand the broader determinants of a particular risk to health.

We began a collaboration between University College London and *The Lancet*: a Commission on managing the health effects of climate change. The main conclusion of this Commission was that “Climate change is the biggest global health threat of the 21^st^ century.”^10^ When we started, it was a fascinating anthropological experience: we brought people together from many different departments in the university who had never met each other before. In our first meeting, there were looks of quizzical astonishment. What were we all doing in this room together? What did we all have in common? When one links together medicine, public health, engineering political science, law, environmental studies, philosophy, economics, and other sciences, one discovers a means to find how we can use the evidence we have generated as scientists to forge a clear path towards a complex social goal.

Let me begin to move closer to where we are today. In 2006, a close friend of mine who is now Dean of the Harvard School of Public Health, Julio Frenk, was Minister of Health in Mexico. Julio called me and said, “Richard, please come to Mexico City. I want to discuss something with you.” The reason he called was that as Minister of Health he had introduced a new set of health reforms called *Seguro Popular*. His goal was universal health insurance. He said, “Richard, the government I’m a part of, led by President Vicente Fox, is about to change and there will be a new president.” That new President turned out to be President Felipe Calderón. “All the work we have done to shape the health reforms in Mexico could be lost in a stroke, because I have no evidence of their successes to pass on to the next government. We have introduced these reforms, but there is nothing in black and white to show the next government what we have achieved. Please work with me to publish a series of scientific papers to demonstrate the successes and the challenges of what we have achieved in Mexico.”

**Figure 6.**
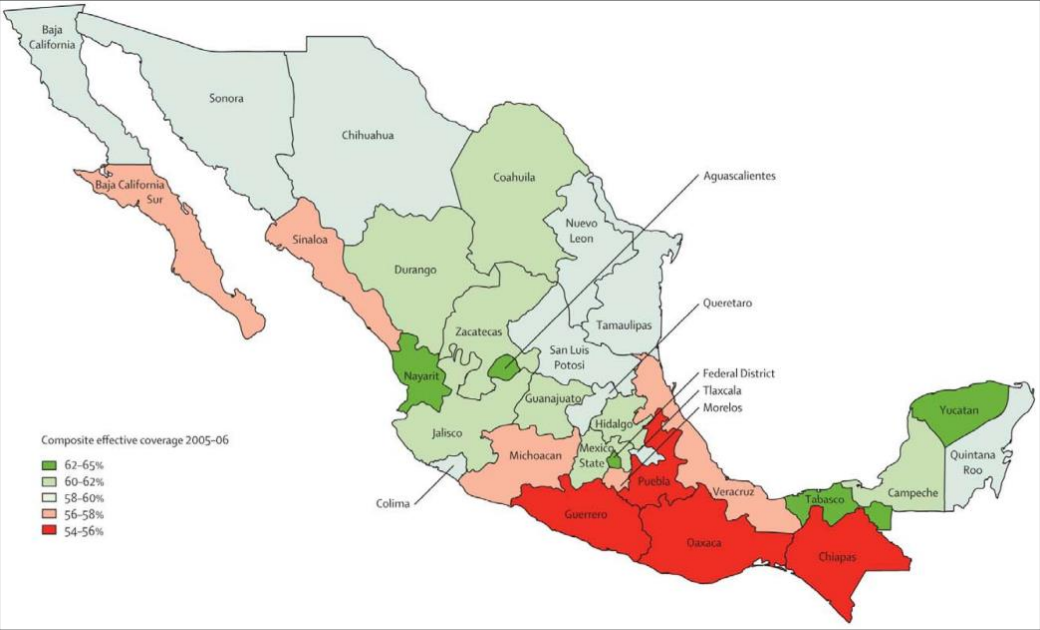
**Mexico’s *Seguro Popular* is an Example of How Politics is Inextricably Connected to Health.**

This was in 2006. I went to Mexico, met with Julio Frenk, we planned this project, and in 2007 we published a series of papers, launched at a symposium in Mexico City with the Mexican government, setting out the evidence about the effectiveness of *Seguro Popular* (Figure 6). This evidence meant that when the government did change the next Minister of Health and the next President could take this series of papers and say, “look at this success, don’t throw it away!” Here was an explicit partnership between scientists, a government, and a scientific journal. We were merely the vehicle, the midwife so to speak, in making sure that the successes, the political successes, of the Mexican health reforms were protected and strengthened in the next government.

**Figure 7.**
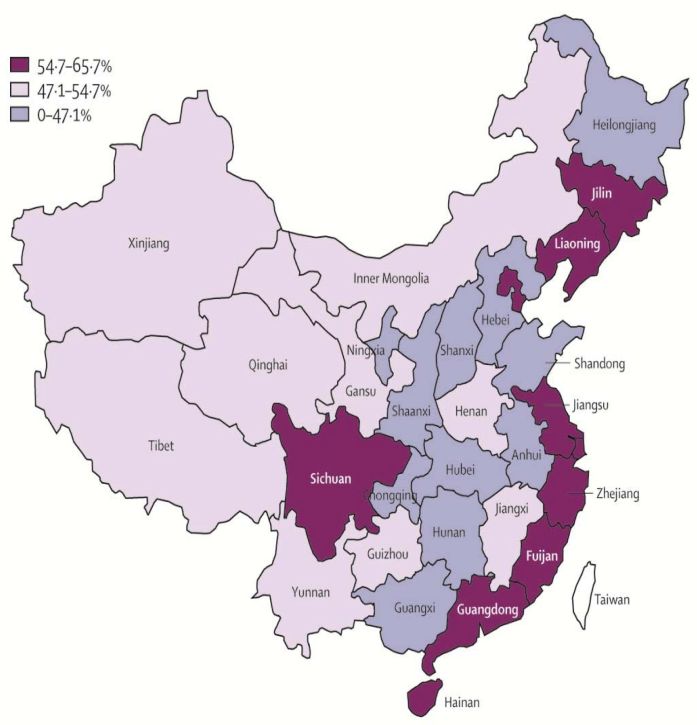
**Health-system Coverage in Chinese Provinces in 2003.**

**Figure 8.**
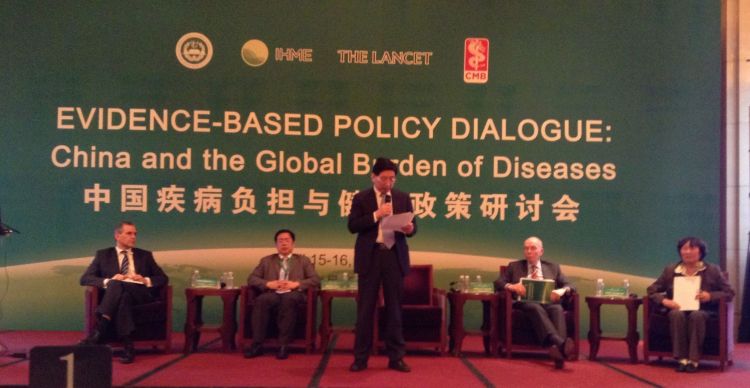
**Evidence-driven Political Reform in China.**

Mexico was quite straightforward. China has been more challenging. China is rightly proud of its achievements. And China does not need the kind of support we offered to Mexico. But health is a critically important issue for China. The government has invested vast sums of money into the health system, higher education, and science as an explicit means to advance the welfare of its people. China faces many difficulties. There are many reasons to be concerned about the political culture in China. But to those who suggest we should boycott China, I say “no”. I do not believe in boycotts. I do believe in partnership, partnerships to create relationships of trust, using science and the values of medicine and health to support a different and better vision for the future. That is what we have tried to achieve in China. We publish a theme issue of *The Lancet* on China every year (Figures 7 and 8). We just held this year’s launch of that theme issue in Beijing last week. And every year we hold a dialogue with the Chinese government where we discuss the latest evidence and progress towards universal health coverage.

Let me reiterate: these political dimensions to health are unavoidable. In a *Lancet* Commission we published in January, 2014, together with the University of Oslo, we looked at the political origins of health inequity, and we investigated how we might use their findings to motivate social and political change.

*The Lancet* has been involved in several difficult controversies over recent years. In 2003, we published a paper on civilian mortality during the Iraq war, and a further paper in 2006 revisiting that same issue. The mailbag I received after publishing the first paper was not at all supportive, to say the least. But what is interesting is how opinions changed. When we published the first paper on civilian mortality^11^, colleagues and friends in the United States were deeply critical of *The Lancet* for getting involved in what they saw as an explicit political issue. “Stop interfering with our government,” was the message I received. That was at a time when President Bush stood beneath a sign saying, “Mission Accomplished.”

By 2006, our early critics had a slightly different view. When we published the 2006 paper on civilian mortality,^12^,^13^ the message I received from friends and colleagues in the United States was different: “Thank you for drawing attention to these concerns.” In the space of just three years, a completely different perspective was apparent regarding ostensibly the same issue. Perhaps this example indicates the potential power of research to challenge but then to bring people together to understand the consequences of political actions on the health of populations.

And just a year ago, we published an open letter on Syria.^14^ from Gro Harlem Burndtland, the former Prime Minister of Norway and Director-General of WHO, drawing attention to an issue of violations of medical neutrality. Open Letters can be valuable and powerful means to emphasise important and neglected issues of general public and political concern.

### THE LANCET IN THE MIDDLE EAST

I had never planned to engage in the political dimensions of health in this region. Every time I was encouraged to write about the occupied Palestinian territory and Israel, I always declined because I had no knowledge or direct experience of this area of the world. Eventually, an invitation did come from Rita Giacaman, who was then Director of the Institute of Community & Public Health at the University of Birzeit. Eight years ago, the Institute was a ramshackle building above a garage in Ramallah. It has since moved to a glittering new home on the main campus of the University. Rita Giacaman, together with an American NGO, Care International, invited me to visit the West Bank, the Gaza Strip, and East Jerusalem to study some of the health predicaments Palestinians faced.

**Figure 9.**
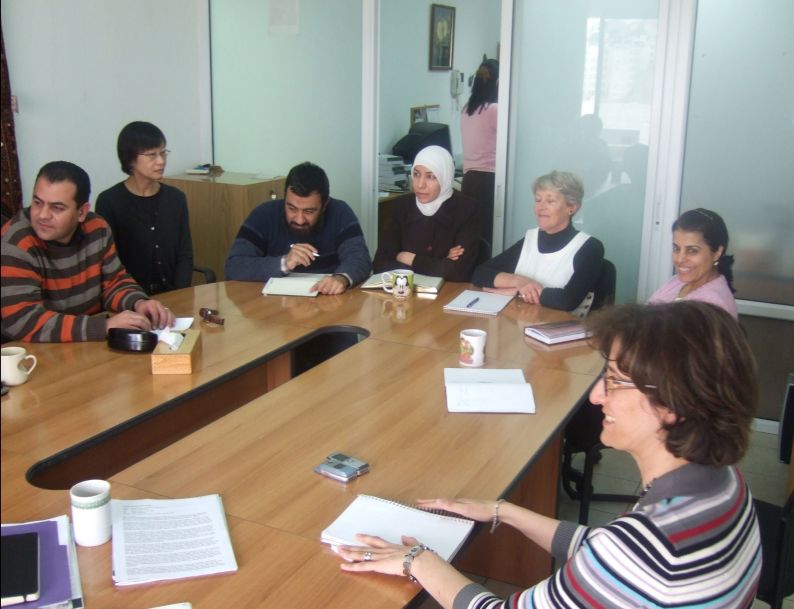
**First Planning Meeting with the Institute of Community & Public Health at the University of Birzeit.**

After that first visit, we said: let us see if we can bring a team of scientists together to tell the story of health in the occupied Palestinian territory. This was our first meeting: sitting at a table in Ramallah to plan what we would do (Figure 9). After two years of work, we published a series of papers examining the health of those living in the occupied Palestinian territory.

**Figure 10.**
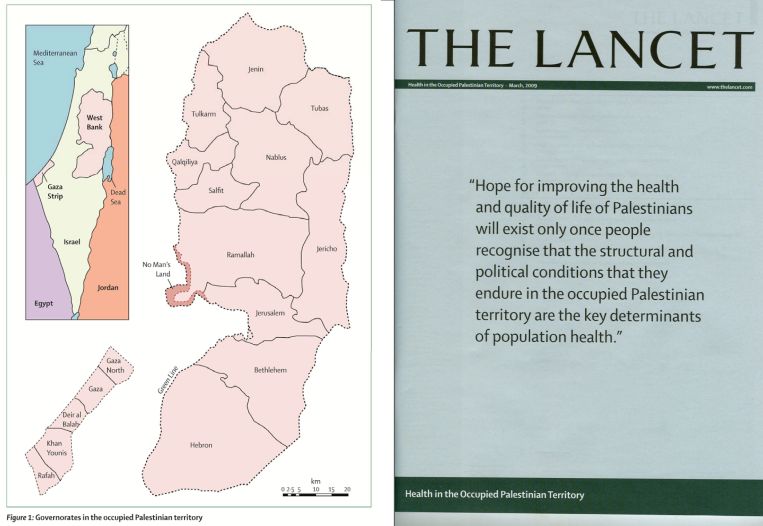
***The Lancet*’s Series on Health in the Occupied Palestinian Territory.**

Here is our opportunity, now in this lecture theatre, in this country. Can we do the same (Figure 10)? Here is the invitation I wish to make today. Our series on the occupied Palestinian territory came from an invitation made by Rita Giacaman. I am here today because of an invitation from Professor Skorecki. I invite you to join with us to tell the story of Israel through health: the successes, challenges, future prospects, the priorities that need to be addressed, and maybe even some ideas for how we could address them together. We have an historic opportunity. Can we seize it together?

The *Lancet* series on Palestine (Figure 10) included five papers. We launched the final report in Ramallah and also in London. Imagine if we worked together on a series similar to this and launched it in Israel and perhaps elsewhere. We have an opportunity to transform the way the world thinks about Israel, its health system, and its relationship with the rest of the world.

When we launched our first series on the occupied Palestinian territory, we had been to Gaza on several occasions, visited people in their homes, observed those same people living their lives, just like you, just like me. They wanted then, and they still want now, to send their children to school, go to work, and do the best they can for their families. The ordinary people of Gaza are not terrorists and they do not represent a terrorist regime—they are people, like you and I, trying to live their lives as peacefully and as safely as they can.

**Figure 11.**
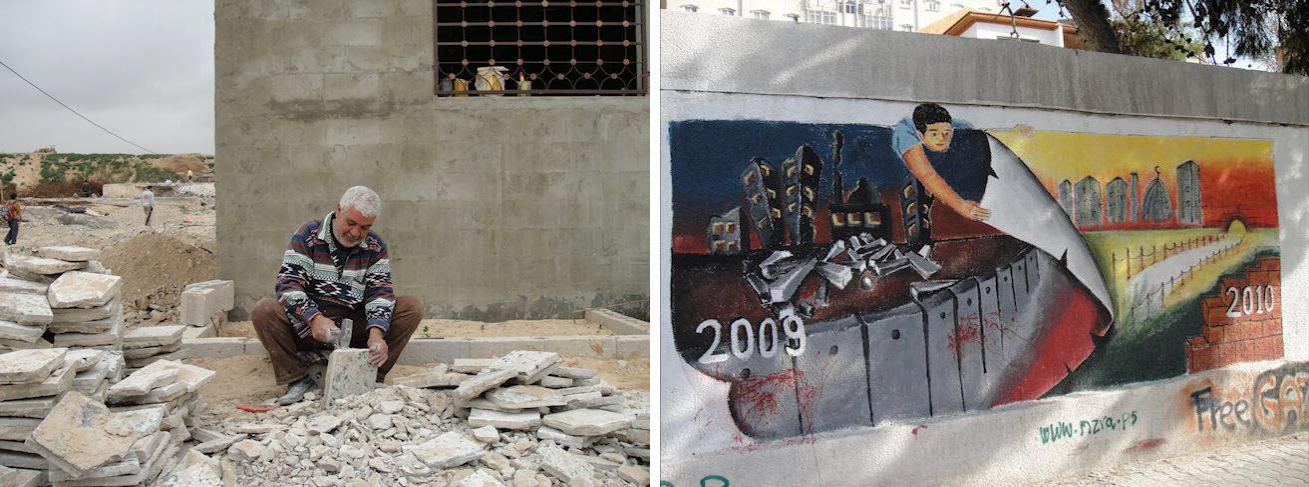
**Photos from a Trip to Gaza.**

When I have been to Gaza, this is what I see. (Figure 11). This is the sentiment that the vast majority of the people of Gaza express. There is hope for a different future, a future of success, prosperity, safety, and peace. The people of Gaza want it. They try to live it. And it is our hope that we can work with them and with you to achieve it.

**Figure 12.**
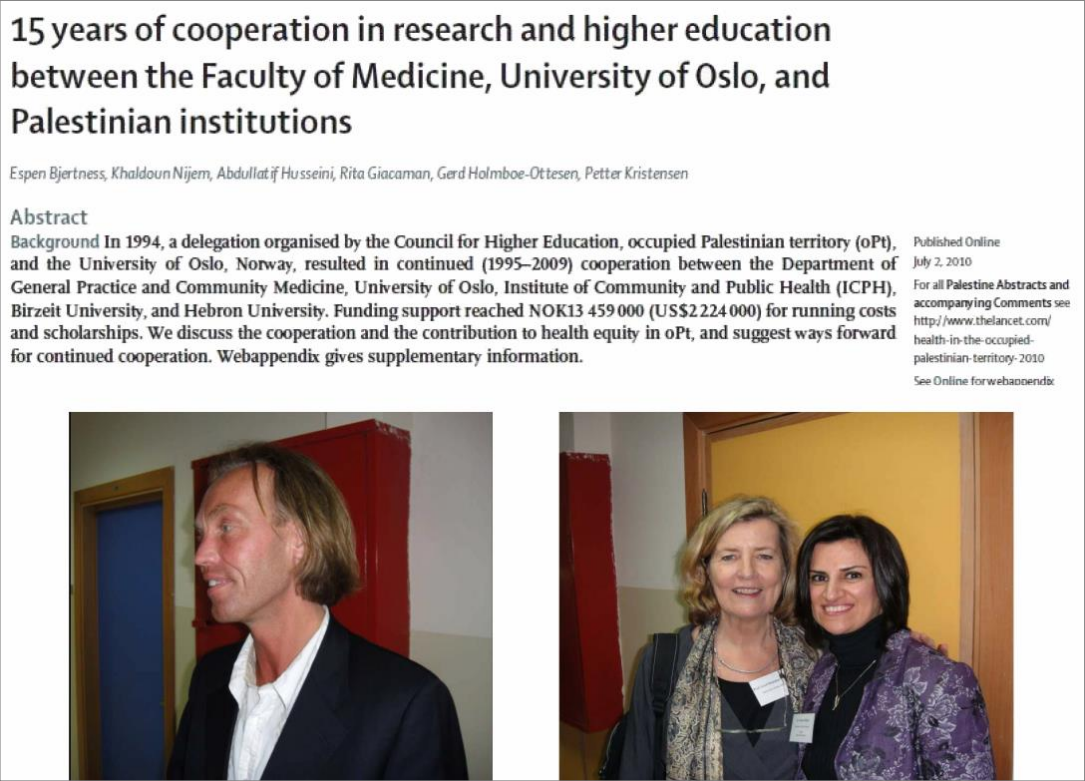
**Cooperation between the University of Oslo and the University of Birzeit.**

The way we have set about this task is through scientific collaboration. Every year *The Lancet Palestinian Health Alliance* meets to bring people together from across the region to present work on the lives of Palestinians, not only in the occupied Palestinian territory but in refugee populations and across the diaspora. Here is an example of cooperation between the University of Oslo and the University of Birzeit (Figure 12)

**Figure 13.**
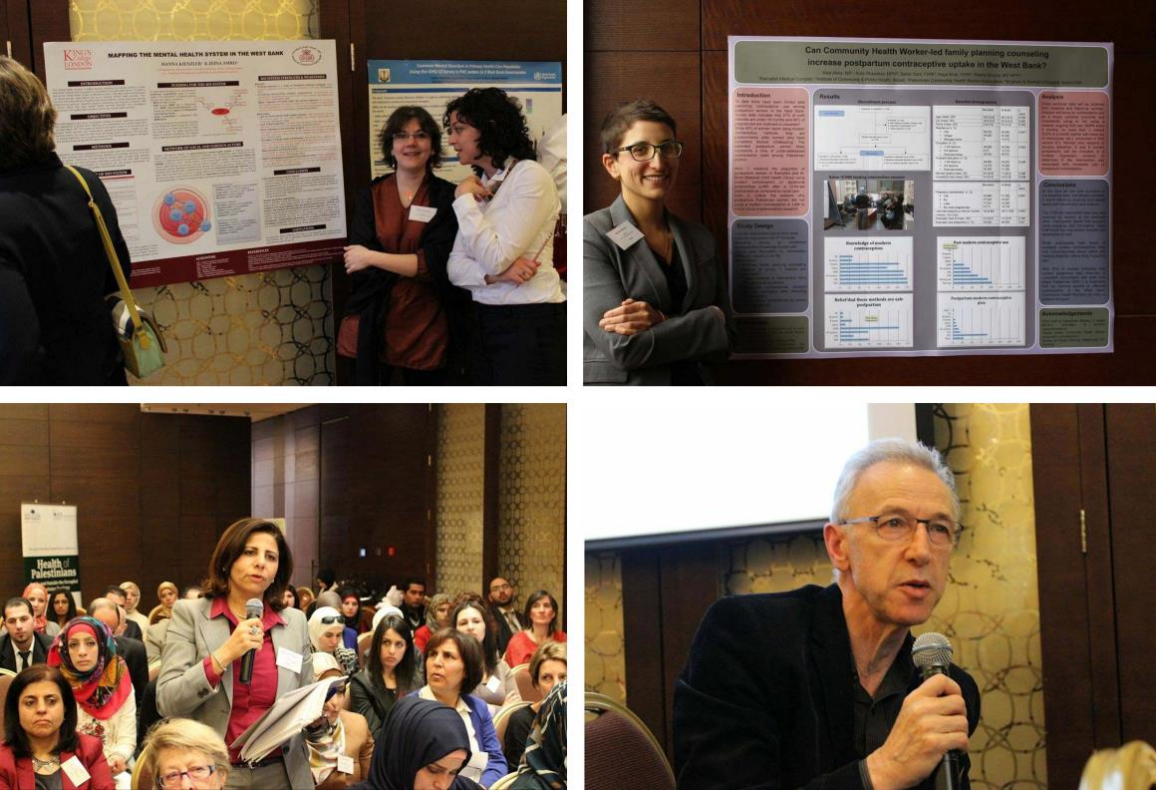
**Collaboration at King’s College on Mental Health in the West Bank.**

The meeting we held this year (March, 2014) was in Amman, Jordan. We are trying to build an extraordinary collaboration. Together with the World Health Organization, UNRWA, universities across the region, an international network of health scientists, and medical charities concerned about the health of Palestinians, we focus on health science and research. There is, inevitably and correctly, a political dimension to this work. The collaboration is about how we can use evidence to advance the case for the health of Palestinian people. It is an exciting meeting. It is about giving a voice to those who typically have no voice. It is about expanding and democratizing the global conversation about health. Here (Figure 13) is a collaboration with King’s College London, looking at the issue of mental health in the West Bank. Or here, reporting on community health and family planning in the West Bank. *The Lancet Palestinian Health Alliance* is a research conference. But it is a research conference with a difference: it aims to use and promote research as an instrument to promote political action in this region.

Another way of viewing our meeting is to see it as a dialogue. We bring people together to question one another. And we bring international academics together to act as independent peer reviewers of the work presented and to ask difficult questions about that work. Two examples: Harry Shannon, from McMaster University in Canada, and John Yudkin, from University College London in the United Kingdom, who come to hold friends and colleagues in Palestine accountable for the quality of their science and scholarship.

Let us imagine we succeed in publishing a *Lancet* series on Israel and its health system. Could we develop our collaboration in a similar way to that of *The Lancet Palestinian Health Alliance*?

Yesterday, I had the privilege of visiting Acco and meeting an Imam and a Rabbi to learn how they work together. At the end of our meeting, and after explaining why I was here, I asked the Imam, “What should I do next?” He said, “You must work with Palestinians. You must work with Israelis. And you must work to bring these two peoples together.”

### CONCLUSION

Last week at the United Nations General Assembly the Assistant Secretary General, Bob Orr, noted that, “There are more crises in the world today than at any time in the history of the United Nations.” What should our response as health professionals be to these predicaments? Let me give two answers, both of which are the same answer, but separated by thousands of years.

In his work *On Obligations*, Cicero wrote this: “First comes that which we see existing in the fellowship of the whole human race…This more than anything separates us from the nature of the beasts.” As modestly as I can say it, this is our philosophy at *The Lancet*—the fellowship of our human species.

This notion was put even more powerfully in the Commission on Human Security, chaired by Sadako Ogata and Amartya Sen, and published at the turn of our new century. Ogata and Sen talked about the importance of “clarifying the need for a global human identity.”^16^ I am half Norwegian and I am half English. I was adopted when I was four months old by an English family. But my father is Norwegian. I only had the good fortune to find him and meet him a few years ago. He was entirely unaware of my existence. Thanks to a DNA test it turned out that he was, indeed, my biological father. But his biology, my biology, did not matter. What matters much more is our shared global human identity.

And scientific journals have an important part to play in promoting the notion of global human identities. As Amartya Sen wrote in his 2010 book, *The Idea of Justice*:

“…an active and energetic media can play an extremely important part in making the problems, predicaments, and humanity of certain groups more understood by other groups.”^17^

What might a journal like the *The Lancet* stand for? Ten years ago, I read a book by a then largely unknown writer to the English-speaking world, Béla Zsolt (his book was first published in 1948). Zsolt was born in Hungary in 1895. He was a writer, a radical social critic, and his family died in Auschwitz. This English translation collected together the edited diaries of his life in the Hungarian ghetto, his life living under fascism in Hungary. In describing that ghetto, he introduces Dr. Nemetti, a medical practitioner who is trying as hard as he can to live his life as normally as possible, despite appalling hardship and oppression. When I first read this book, my heart skipped a beat. There is one sentence that stopped me in my tracks—recall, this man, this doctor, desperately trying to live a normal life in extraordinarily abnormal circumstances:

“Perhaps because outside he had been a passionate doctor, always studying, keeping up with international developments, reading the *Münchener Medizinische* and *The Lancet*.”^18^

Here was a remarkable connection. Between a doctor living under threat of genocide and doctors today, including myself now as Editor of that same journal which gave him some sense of continuity through a time of terrifying existential destruction.

*The Lancet* was founded on the principle of advancing equity between peoples. Put in more modern terms, the right to the highest attainable standard of health, progressively realized everywhere and for all. We are a journal that stands for life, for survival, for resistance, and for resilience. For human flourishing, for human fellowship, and for our responsibility to each other. We stand for hope and we stand for opportunity. We stand for the positive power of science and medicine to shape and change our futures. With those principles and values in mind, I hope we can join together today for a fresh start towards a new and different future.

Thank you.

## Abbreviations:

CDC: Centers for Disease Control Prevention;
WHO: World Health Organization.
